# Supplementary material for: Promoting Neuronal Outgrowth Using Ridged Scaffolds Coated with Extracellular Matrix Proteins
Source: Biomedicines. 2021 Apr 27;9(5):479. doi: 10.3390/biomedicines9050479 (PMC8146557; doi:10.3390/biomedicines9050479)
Supplement: Supplementary file 1 [file biomedicines-09-00479-s001.zip › biomedicines-1179506-supplementary.pdf]

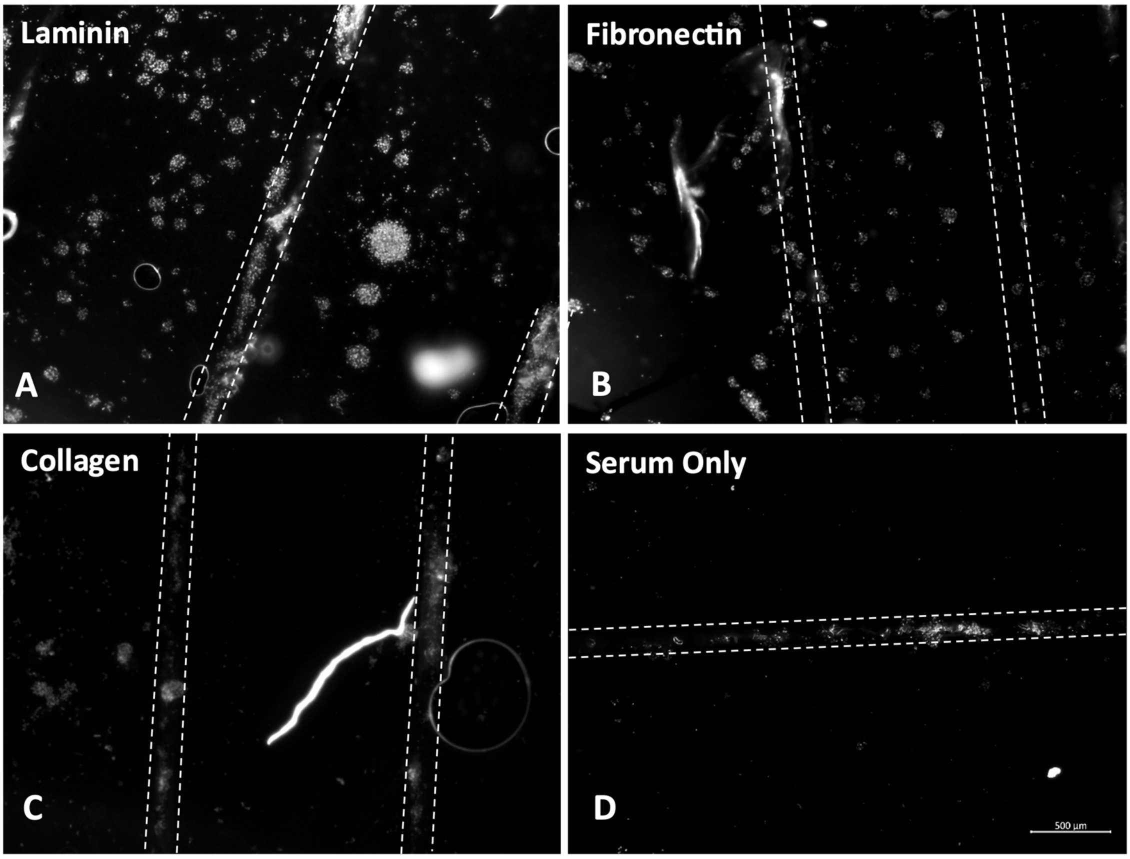

Figure S1. DAPI staining of dissociated DRG neurons on OPF+ scaffold sheets with ridges 1 mm apart. The sheets were coated with (**A**) Laminin, (**B**) Fibronectin, (**C**) Collagen, and (**D**) Serum only. Number of cells on and off ridges was determine by DAPI co-labelled with b-III tubulin (Figure 3).
